# Sea of plastic: representations of the sea and pro-environmental attitudes and behaviors regarding marine plastic pollution in Peru and Chile

**DOI:** 10.3389/fpsyg.2023.1308796

**Published:** 2024-01-18

**Authors:** Fernanda Saavedra, Marisol Mego, Sofia Ticona, Martin Thiel, Jostein Baeza, Agustín Espinosa

**Affiliations:** ^1^Grupo de Psicología Política y Social de la Pontificia Universidad Católica del Perú, Departamento de Psicología, Pontificia Universidad Católica del Perú, Lima, Peru; ^2^MarineGEO Program, Smithsonian Environmental Research Center, Edgewater, MD, United States; ^3^Facultad Ciencias del Mar, Universidad Católica del Norte, Coquimbo, Chile; ^4^Center of Ecology and Sustainable Management of Oceanic Island (ESMOI), Coquimbo, Chile

**Keywords:** marine plastic pollution, representations of the sea, pro-environmental attitudes, pro-environmental behaviors, anthropocentrism

## Abstract

Marine plastic pollution remains one of the greatest problems worldwide. Hence, this study explores the attitudes and pro-environmental behaviors of Peruvian and Chilean citizens regarding marine pollution, with an emphasis on plastic pollution. For this, forty-four semi-structured interviews were conducted with Peruvian (*n* = 24) and Chilean (*n* = 20) citizens, of which, through thematic analysis, semantic patterns were identified. Results show that the participants’ representation of the sea is positive and related to the connection they report having with this environment. Additionally, it was found that the sea fulfills recreational and economic purposes, reflecting an anthropocentric perspective, since it is associated with leisure and resource extraction, respectively. Both purposes are related to the causes of plastic pollution, although with differentiated effects. Anthropocentrism is also reflected in the direction that environmental concern takes towards the impact of this type of pollution predominately on individuals and society. Regarding pro-environmental behaviors, most of the initiatives proposed by the participants in response to marine plastic pollution correspond to individual actions, which could be due to the fact that they perceive a low commitment level from authorities to address the problem. In particular, the Chilean participants attributed a greater role to their authorities in dealing with plastic pollution, which would indicate a more institutional perspective of the problem. Thus, it is proposed that to address marine plastic pollution it is necessary to articulate individual actions with public policies carried out by social stakeholders such as governments, companies and non-governmental organizations, in order to build a more efficient culture of marine protection.

## 1 Introduction

The study of environmental issues has taken two approaches to the relationship between humans and nature: environmentalism and ecologism. The difference between both is that environmentalism has an anthropocentric perspective on the environment, i.e., concern for nature revolves around the needs and interests of humankind (Ibarra, 2009; Hoffman and Graham, 2015). In contrast, ecologism has an ecocentric perspective, since it postulates that nature has an intrinsic value, positioning humans as one more element of nature (Hoffman and Graham, 2015).

One of the major discussions in environmentalism and ecologism takes place in the context of the environmental crisis, characterized by an interrelated set of specific problems such as depletion of natural resources, different forms of environmental pollution, decrease of biodiversity, etc., (Hoffman and Graham, 2015). This environmental crisis is related to the effect that human behavior has on the environment at different scales, like changes in production and consumption patterns and other processes exacerbated by globalization (Foladori, 2001). These effects, in turn, have resulted in risks for humans and ecosystems (Foladori, 2001; Intergovernmental Panel on Climate Change (IPCC), 2014).

The marine ecosystem, among others, fulfills economic and psychological functions (Armstrong, 2020). At the economic level, it is a supply of food and other natural resources obtained from extractive activities (Intergovernmental Panel on Climate Change (IPCC), 2014; Saavedra and Mardones, 2021). At the psychological level, it is a source of wellbeing that allows the expression of cultural and aesthetic manifestations, provides spiritual wellbeing and stimulates recreation (Intergovernmental Panel on Climate Change (IPCC), 2014; Thushari and Senevirathna, 2020). These functions indicate the predominance of humanity’s instrumental view of the sea. From the perspective of environmental psychology, the more dominant this instrumental approach is, the greater the potential for endangering the ecosystem (Uenal et al., 2022).

This instrumental view of the sea might be related to the predominance of neoliberalism in Peru and Chile during the last decades (see Rottenbacher and Schmitz, 2012). Neoliberalism is an economic and political system that reduces government intervention in favor of the free market, placing producers and consumers in charge of regulating the system (Guillén-Romo, 1992). Under this system, the government ensures the right conditions to maintain macroeconomic growth, even at the expense of the people and the environment (Rottenbacher, 2010; Geiger et al., 2022).

Thus, some of the social consequences of neoliberalism are economic growth at the expense of increased inequalities and a society with individualistic and depoliticized values (Rottenbacher and Schmitz, 2012). These, in turn, justify the system with attitudes, values and practices that prioritize individual and interpersonal interests over the public one (Rottenbacher, 2010). Hence, the environmental crisis facing the marine ecosystem goes unnoticed at the political, economic and social levels due to a system that makes it invisible or delegitimize it (Geiger et al., 2022).

The influence of social and economic systems, such as neoliberalism, on environmental concern might be understood through, what the New Ecological Paradigm (NEP) identifies as, the two dimensions at the basis of environmental attitudes and behaviors: ecocentrism and anthropocentrism (Dunlap et al., 2000). From an ecocentric perspective, environmental protection is important for its intrinsic value, transcending the capacity of natural resources to satisfy functions in favor of humanity (Thompson and Barton, 1994). In that sense, people with an ecocentric perspective, following biospheric values, will choose to perform pro-environmental actions based on the cost and benefit for the environment (Steg and de Groot, 2012). In contrast, the anthropocentric perspective argues for the protection of nature based on the value of natural resources and ecosystems in maintaining and improving quality of life (Thompson and Barton, 1994). This anthropocentric dimension is aligned with selfish and altruistic values. Those who follow selfish values will decide to perform pro-environmental behaviors considering costs and benefits for themselves, while those who prioritize altruistic values will take into account the repercussions that marine pollution has on other individuals or society (Steg and de Groot, 2012).

In relation to this, environmental attitude incorporates cognitive, affective and dispositional components, characterizing an opinion or predisposition to act in favor of the environment (Páramo, 2017). In this sense, people’s opinion about environmental protection and natural resources precede environmental behavior (Torres-Hernández et al., 2015). The cognitive component refers to the knowledge and information about environmental problems, their causes and the mechanisms to avoid and correct them. The affective components are psychological factors such as feelings or attitudes of concern for environmental conservation. The dispositional component includes personal attitudes towards pro-environmental action and the cost of implementing environmental policies (Jiménez and Lafuente, 2010).

Associated with environmental attitudes, the connection with nature is a cognitive and emotional connection based on affective experiences associated with staying in nature (Perkins, 2010). While being in connection with nature, love and concern for it involve a sense of responsibility and commitment to environmental protection (Wu and Zhu, 2021). In that sense, the greater the connection with nature, the more likely it is that pro-environmental behaviors will be performed (Davis et al., 2009).

**Figure 1:**
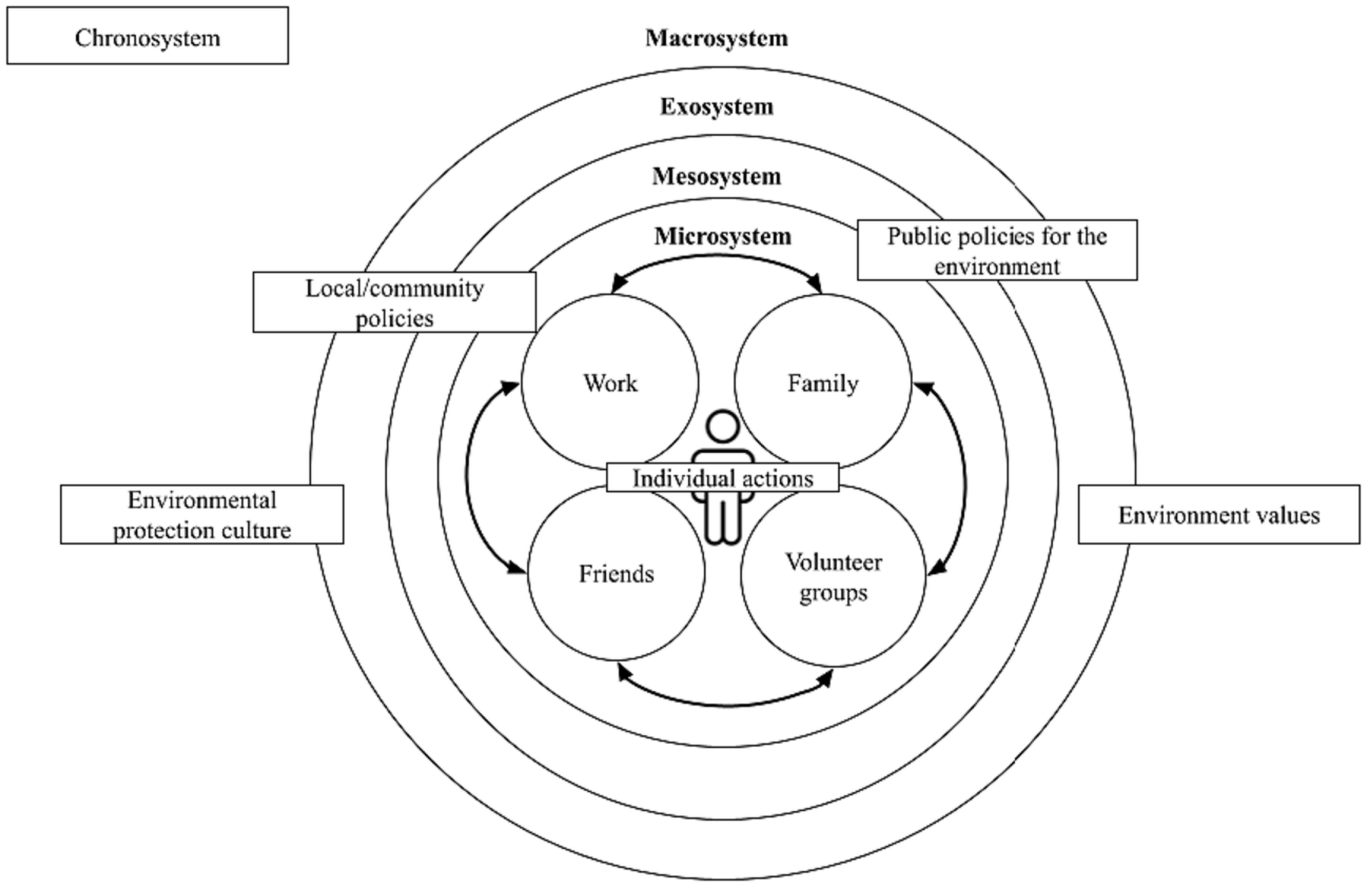
Socialization of pro-environmental behaviors. Elaborated by the research team. Based on Hoffman and Graham’s levels of analysis of pro-environmental behavior and Bronfenbrenner’s ecological theory.

Pro-environmental behavior comprises deliberate and effective actions aimed at protecting the environment (Páramo, 2017; Rivera-Torres and Garcés-Ayerbe, 2018). However, despite the wide acceptance of the research of environmental attitudes, several studies report inconsistent results regarding a statistically significant relationship between attitude and pro-environmental behavior (Newsome and Alavosius, 2011; Páramo, 2017; Olivera et al., 2020). This might be linked to the weighting of values, such as competition and individualism, within society that influences selfish attitudes (Corral et al., 2006). However, from a more ecological perspective of pro-environmental behavior, four levels of analysis are proposed to explain the different range of involvement, from individual action to cultural representation (Hoffman and Graham, 2015). It is proposed that the levels of pro-environmental behavior could be understood as expressions of a model of socialization of environmental attitudes and behaviors. This is based on Bronfenbrenner’s (1979) ecological theory, which states that human socialization occurs in the interaction between the different systems humans engage with (Figure 1).

Based on what Hoffman and Graham (2015) proposed, the first level of pro-environmental behavior involves individual actions of people committed to the environment, such as cleaning up litter, segregating waste, using reusable products, etc. These pro-environmental actions occur within systems people belong and interact with, such as family, work peers, friends, or volunteer groups; this is known as microsystem (Bronfenbrenner, 1979).

These systems, although independent of the individual, are influenced by them and vice versa (Bronfenbrenner, 1979). For example, if the individual lives with a family that promotes pro-environmental behaviors, they are more likely to develop environmental protection habits. Similarly, individuals who volunteer to promote the use of non-plastics are more likely to reduce their plastic consumption, and their actions could influence social groups close to them. According to Bronfenbrenner (1979), these systems in which the individual participates within the microsystem can be connected to each other through a network that constitutes the mesosystem. For example, a plastic bottle recycling campaign at work encourages collaborators and their families to participate in the activity.

The second level of pro-environmental behavior is constituted by the particular policies implemented at the local level in favor of the environment (Hoffman and Graham, 2015). These policies are implemented in settings where the person does not actively participate but is affected by occurring events; this is defined as exosystem (Bronfenbrenner, 1979). Particular policies are issued by authorities, policymakers and people in power who have an impact at the local level. These institutions form systems that influence people’s pro-environmental behavior although they might not actively participate in. For example, municipal ordinances, although established by local authorities, regulate the environmental behavior of individuals even if they have not been directly involved with it.

The third level refers to a set of public policies promoted by an interest group (Hoffman and Graham, 2015). This level, like the previous one, also belongs to the exosystem. The difference is that these policies have a wider scope such as national, regional or international. For example, the consumption tax on plastic bags has led stores to apply a fee to customers for plastic bags (Xanthos and Walker, 2017), promoting sustainable alternatives.

The fourth level refers to the society’s cultural proposal of prioritized values (Hoffman and Graham, 2015), such as pro-environmental values and environmental protection. These cultural or ideological elements are human-related and have influence on lower systems, hence, as a group, they form the macrosystem (Bronfenbrenner, 1979). Consequently, a society that promotes ecological attitudes and behaviors would build a citizenship culture that fosters environmental protection, expressed in the demand for environmental policies and the promotion of sustainable habits.

In the present study, we explored the relationship of these levels of analysis and socialization of pro-environmental behaviors in marine environments, whose physical, chemical and biological properties have been altered by global warming and pollution (Intergovernmental Panel on Climate Change (IPCC), 2014). The main problems affecting this ecosystem are the warming and acidification of its waters, destructive fishing practices, petrochemical exploitation of the seabed and plastic pollution (Armstrong, 2020; Banco Mundial, 2022).

Plastic pollution in the sea is an alarming global problem caused by human behavior at the individual, collective and societal levels (Pahl et al., 2017). Some sources of plastic pollution are industrial, recreational or economic activities (Bravo et al., 2009). Another source of plastic comes from solid waste generated on land and offshore, that decomposes into small particles: microplastic, which can generate the death of marine species and alter the marine ecosystem (Fernández and Jiménez, 2020; Paredes-Osses et al., 2021; Smith and Brisman, 2021; Ortiz-Alvarez et al., 2022). Furthermore, marine plastic pollution has an impact at the social and economic level. At the social level, there are negative consequences on human health related to food safety and exposure to chemical substances due to microplastics in the food chain (Aretoulaki et al., 2020), on people’s lifestyles, mental health, identity and cultural heritage (Yose et al., 2023). At the economic level, there is a loss of income associated with marine and coastal activities such as fishing, aquaculture and marine tourism, due to the high costs of remediation activities such as beach cleanup (Newman et al., 2015; United Nations Environment Programme, 2021).

In this context, Peru and Chile might be two key countries involved in marine plastic pollution since, in 2017a new plastic island near the coasts of both countries was found (Sierra, 2018a). In those lines, the most common products found on these coasts are single-use plastics such as bottles and plastic bags (Gómez et al., 2020; De-la-Torre et al., 2021). Studies in Peru and Chile have reported that part of the plastic pollution comes from economic activities such as fishing, since the greatest polluting source is abandoned, lost or discarded fishing gear (Araya-Schmidt and Queirolo, 2019; Deville et al., 2023). Lost gear traps and attracts different animals, which can lead to their death and the continuation of a cycle of entanglement (Ryan, 2018), and disperse invasive species altering the marine ecosystem (Ferrigno et al., 2018). Plastic pollution might have a terrestrial origin by sewage spills, polluted river basins, etc., (Bravo et al., 2009; Ita-Nagy et al., 2022), and a marine origin by the consumption of food and beverages on boats, the loss of equipment, products for boat maintenance, among other sources of microplastics (Deville et al., 2023).

Thus, marine plastic pollution might be one of the most environmental problems these countries face. A global poll identified that Peru and Chile were two countries that recognize the importance of a plastic global treaty the most in the region. While Chile supported more the prohibition of single-use plastic than Peru, the latter was involved in a proposal to the United Nations to consider the role of the life cycle of plastics in environmental pollution (World Wide Fund for Nature, 2022).

Nonetheless, in Peru, plastic remains one of the most common sources of marine pollution (De-la-Torre et al., 2021; Ita-Nagy et al., 2022; Deville et al., 2023), despite the existence of public policies such as Law No. 30884 (El Peruano, 2018) which, as of 2018, prohibits the consumption of such materials in protected natural areas. Despite its approval, Congress claimed that part of the law, specifically the ban on the manufacture of expanded polystyrene (EPS), affected small and medium-sized companies and should therefore not be implemented (Morales, 2022). This institutional discrepancy might demonstrate the authorities’ lack of interest in environmental issues.

In the case of Chile, in 2021, Law No. N.° 21,368 was passed, banning the free service of single-use plastic items in stores throughout the country, limiting the supply of plastic items, and promoting environmental education about the ecological impact of single-use plastic engaging companies, municipalities and the Ministry of Environment (Congreso Nacional de Chile, 2021). Years before, the establishment of fines for littering in beaches and riverbanks were approved (Urbina et al., 2020). These actions were possible due to the growing concern that governmental institutions and non-governmental organizations were having about marine litter, which influenced the involvement of multiple municipalities in reducing the consumption of single-use plastics (Cristi et al., 2020). The success of such efforts also depends on the actions of citizens, which may be affected by a given context. In this regard, a study conducted in coastal regions of Chile found that those who are more willing to engage in pro-environmental behaviors valued their cultural history, felt a connection with nature, and their regions had an economy based on sustainable tourism. This was not the case in regions whose economy was based on mining or aquaculture, and with few spaces to enjoy nature (Kiessling et al., 2017).

Despite these advances, there is discontent among Chileans, manifested in massive protests, towards their political institutions, as environmental issues have a direct impact on the residents of regions where extractive activities predominate (Vera, 2017; Allain, 2019). This is consistent with several studies that report that when people feel connected to an ecosystem, they tend to engage in pro-environmental behaviors (Daryanto and Song, 2021). However, as identified in the contexts of Peru and Chile, when such preservation measures affect the interests of large companies, the protection of ecosystems is given low priority (Kiessling et al., 2017; Morales, 2022).

Based on all the above, this study aims to contribute to the knowledge about representations of the sea and the understanding of pro-environmental behavior as a product of a socialization process, taking Bronfenbrenner’s ecological model as a reference. In addition, it seeks to highlight the structural dimension of pro-environmental behavior while identifying its individual and collective levels of action. Therefore, the general objective of this study is to explore the attitudes and reported-pro-environmental behavior of Peruvian and Chilean citizens regarding marine plastic pollution. To this end, three research objectives were proposed: (1) explore the representations that citizens of both countries have about the sea and the problems associated with this ecosystem; (2) identify the causes and consequences that citizens of both countries recognize of marine plastic pollution; and (3) learn about the actions that citizens of both countries propose to take to reduce marine plastic pollution.

## 2 Methods

### 2.1 Participants

Forty-four people participated, 24 Peruvians (16 women and 8 men) between 18 and 54 years old, and 20 Chileans (15 women and 5 men) with ages between 22 to 60. In the Peruvian case, participants reside in Metropolitan Lima, except for one from Lambayeque. In the Chilean case, they live in the regions of Atacama, Coquimbo, Valparaíso and Santiago Metropolitan. Most of them have direct contact with the sea, and visit it between a daily and yearly period (see Supplementary material).

The inclusion requirements were to be over 18 years old, reside in one of the countries involved in the study and visit the sea to a greater or lesser extent. The interviews were conducted remotely by the Peruvian team. Participants from Peru and Chile were recruited through a closed-ended recruitment of convenience and an open call through social media, respectively. In the Peruvian case, the team directly called contacts that matched the profile via online messaging, due to easy access and availability (Hernández et al., 2014). Information was provided on the characteristics of the study and confirmation of participation was requested.

Due to the fact that interviews were to be conducted remotely by the Peruvian team, for the recruitment of Chilean participants, the research peers in Chile, from the Universidad Católica del Norte, were in charge of broadcasting the study through their social media, indicating its objective and the participants’ profile. Those interested were asked to fill in a registration form to confirm that they met the requested profile. Subsequently, registrants were contacted by the Peruvian Tema via email to provide more information about the characteristics of the study and to confirm their participation. In both cases, the saturation criteria was used to determine the final number of participants, since it allows stopping the data collection when no more categories emerge to be analyzed (Creswell et al., 2007). The coding of the participants consisted of the country of origin (Peru or Chile) and the number of the interview.

Regarding ethical aspects, an informed consent protocol was used to indicate the objective of the study, the duration of the interview (45 min), and the participation conditions. In agreement with the principle of participant autonomy, the voluntary and confidential nature of participation was emphasized, guaranteeing the protection of the participant’s identity by placing a code when transcribing the interviews and presenting the results. In addition, permission was requested to audio-record the interview, noting that it would be used only for academic purposes and handled only by the research team in a cloud storage system. On the other hand, in compliance with the principle of beneficence and nonmaleficence, it was reported that participation would not represent any harm or damage, since the study had minimal risk. Finally, it was indicated that the return of results would be given through the social media of the research groups involved, and the e-mail address of a member of the research team was provided in case there were any doubts about the study.

### 2.2 Data collection

A semi-structured interview guide was prepared to facilitate dialogue with the participants, a focused analysis of the details of the discourse, and delving into new and relevant information (Perpiñá, 2012; Hernández et al., 2014). Its structure was based on three thematic axes, with a total of ten main questions with their respective sub-questions (see Table 1). The interview guide was elaborated based on the literature review and the study’s objectives.

**Table 1:** Interview’s thematic axis and main questions.

Thematic axis	Main questions
Axis 1. To explore the representations that Peruvian and Chilean citizens have about the sea	For you, what is the sea? How important is the sea for you? Why? How important is the sea for your activities? How do you feel when you are inside or around the sea? Why?
Axis 2. Identify the problems that Peruvian and Chilean citizens recognize in the sea	Since you started going to the sea, what changes have you noticed throughout these years? What do you think are the main problems affecting the sea? What do you think about plastic pollution in the sea? Do you know about microplastic pollution in the sea?
Axis 3. To learn about the actions that citizens in Peru and Chile propose to take to reduce plastic pollution in the sea	Do you know of any measures that are being taken to reduce plastic pollution in the sea? What do you think about it? What do you think could be done to reduce plastic pollution in the sea?

### 2.3 Procedure

After recruiting the participants, the interview’s time and date were coordinated through the Zoom platform. Before each interview, the informed consent protocol was implemented. The interviews lasted approximately 45 min and were conducted in two phases. Fieldwork was conducted in Peru from January to August of 2022, while in Chile from April to October of the same year. Once data saturation was reached, the recruitment was closed and the interviews were transcribed. Finally, the information from both countries was analyzed. The overall procedure, including the data collection and the information analysis was carried out in Spanish. For the purpose of this article, the results and final report of the study were translated to English.

### 2.4 Information analysis

The analysis of the information consisted of a thematic analysis, the aim of which was to identify the most relevant and frequent patterns of meaning in the participant’s discourse. This procedure was carried out in three stages using Atlas.ti 9 software. The first stage was open coding, this served to identify the content of the interviews (Scribano, 2000) systematically and objectively. Thus, analysis codes were obtained and systematized in a codebook (see Supplementary material). These codes were established since an inductive logic, that is the identification of themes from the analysis of the participant’s discourse that accounted for their feelings and experiences (Hernández et al., 2014). Once some families of codes were identified, axial coding was carried out in parallel, which sought to link the codes according to their dimensions and properties around categories representing thematic axes (Strauss and Corbin, 2002). During the coding process, the saturation of the information was considered, which marks the moment when, after analyzing multiple interviews, new information cannot be found and, thus, the coding analysis concludes (Hernández et al., 2014). Finally, the categories were analyzed through the revised literature and reviewed by the research team and other experts in the field.

## 3 Results

### 3.1 Representations of the sea: influences of recreational and economic use

**Figure 2:**
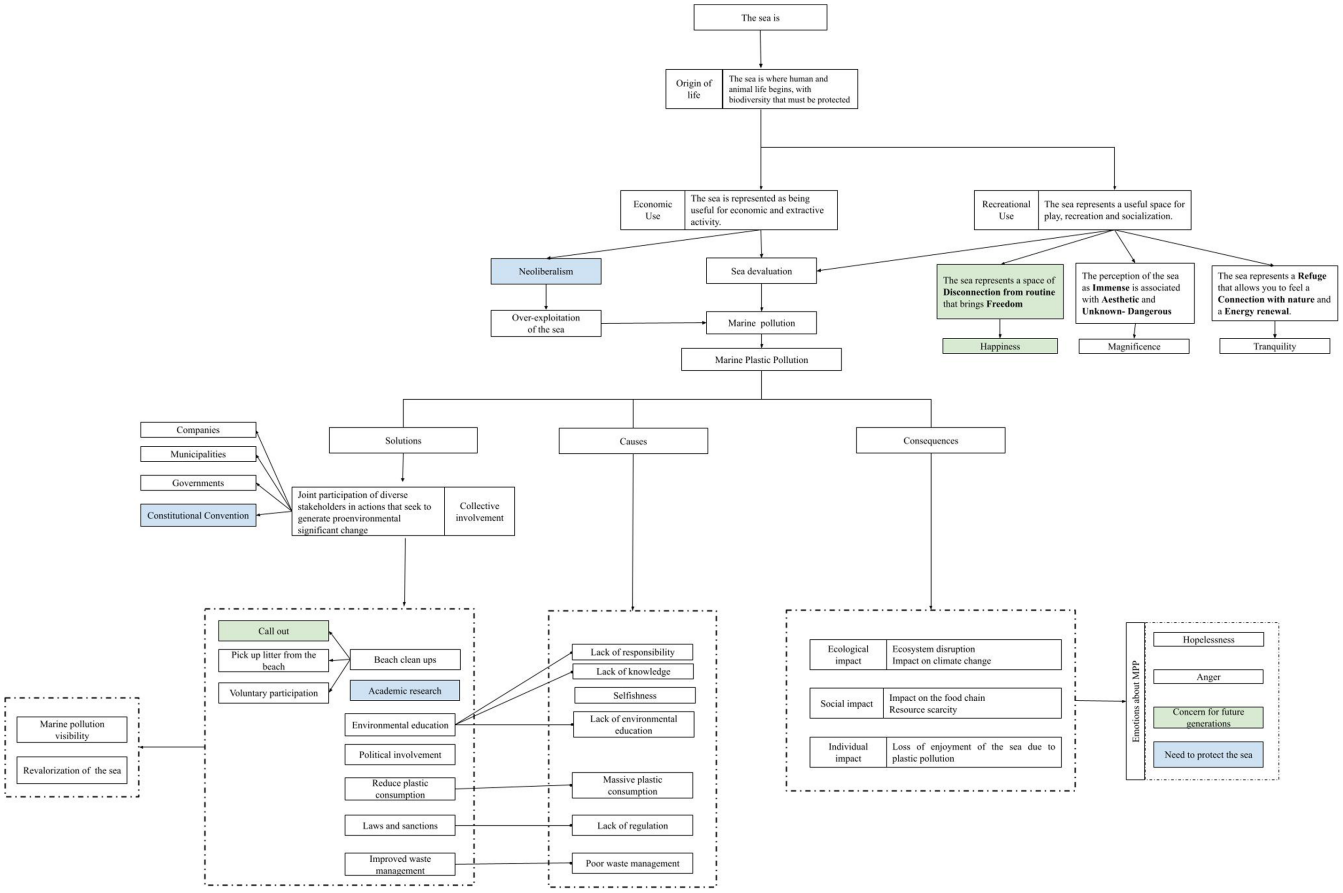
Code map of representations of the sea, attitudes and pro-environmental behaviors regarding marine plastic pollution. Green colored codes indicate that it was identified predominantly in Peruvian participants and sky-blue colored codes indicate that it was identified predominantly in Chilean participants. Transparent colored codes represent evenly participants of both countries.

The Peruvian and Chilean citizens’ representation of the sea is characterized mainly, at the cognitive and affective level, by a positive appreciation of this environment (see Figure 2). This might be related to the fact that most of them had frequent contact with this ecosystem (see Supplementary material). According to the participants, the sea is the origin of life, a space that allows human life and where a wide biodiversity resides. Thus, the sea is represented as “a great source of oxygen, food and recreation for humans” (Participant Chile 14). Based on this, the participants attributed two main purposes to the sea: recreational and economic use.

Most of the participants highlighted the recreational use, the perception of the sea as a space for sports, games and social interaction. According to the Peruvian participants, who reported this purpose most often, “most people go to the sea to swim or drink beer. They see it as a recreational space” (Participant Peru 17). This brings them happiness, which is described as a feeling of exaltation, wellbeing and fun: “a feeling of pleasure, one of the greatest pleasures I feel is when I run, jump into the ocean and swim” (Participant from Chile 11).

Furthermore, this recreational use is often associated with the feeling of connection with nature, understood as the feeling of being connected with the sea, the life that converges in it and with oneself. For some participants, this connection is experienced as a mimicry with the sea, and for others it is “a connection with yourself, you discover yourself in the water” (Participant Chile 6). In this way, the participants recognize the sea as a refuge: a safe and appropriate place to relax and express emotions. In this sense, having access to the sea produces tranquility in most of the participants from both countries, understood as the feeling of not having problems or worries: “… it is something very comforting, if I am stressed or worried about something, the marine environment relaxes me” (Participant Chile 10). The association between this representation of the sea with the feelings of connection with oneself, security and relaxation generate in them respect for this environment: “I think it is a safe space for people, the feeling of being isolated when you are in the sea is soothing and relaxing. This can give you a great respect for the sea, to understand that it is the only place where you are alone, where you can be safe and you can do what you like” (Participant Peru 5).

In this way, for some participants the sea represents a space for disconnection from routine, to get away from work and the city: “those of us who study, work, feel stressed in the day-to-day feel that the sea is like a balance that takes you out of the routine and recharges you with energy” (Participant Peru 6). This energy renewal is described by the participants as a return to a sense of well-being. In addition, the disconnection from routine produces freedom in most participants from Peru and Chile, the transitory state of having no restrictions or responsibilities of one’s own free will. Moreover, the sea acquires value for what it offers to people: “[the sea] is an environment for anyone who wants to take a space to renew themselves, to recharge their energy, to de-stress, to free themselves. Also, I think it is an invaluable space for society” (Participant Peru 12).

To some Peruvian and Chilean participants, the importance of the sea is associated with aesthetics, the perception of the sea as something beautiful and pleasant to observe: “for me it is significant and beautiful, the colors, the sun, the clouds, the wind, the movement is different, it is wonderful” (Participant Chile 13). This appreciation of the sea comes from their own definition of magnificence, the feeling of witnessing an immense element that surpasses their own capacities: “I recognize the strength and power it has, and that means that I recognize my human weakness. It is about respecting that there is something bigger” (Participant Chile 14). The respect for the sea is also inspired by its representation as an unknown element, which cannot be controlled, and as a potentially dangerous place for people, which cannot always be trusted: “I believe that nature cannot be challenged, because when it wants to show itself, it does. I am not going to face a rough swell, I do not go near if there is a high tide, because it is said that the sea is treacherous” (Participant Chile 8).

However, in opposition to respect, recreational use is also linked to the exploitation of the sea, understood as the low appreciation and low prioritization of marine protection by the general population. For the participants this is because “in the minds of people the sea is a place where you can go, enjoy and be happy. There is no sense of marine protection” (Participant Peru 11). There is a perception that the lack of concern about marine protection might be related to a limited representation of the sea as only a source of wellbeing and/or economic profit: “I think that, first, as humans, as a population, we are mainly responsible for not having been able to protect our sea, first because we think that it is only a source of economic benefit or aesthetic-visual attraction, but we were never educated in its importance” (Participant Chile 11).

In this way, the exploitation of the sea could also be associated with its economic use which consists of economic and extractive activities. In this sense, the sea “is a work device…, thanks to this, we can have a development of economic progress” (Participant Chile 7). For some participants, mostly from Chile, this representation of the sea could be influenced by the neoliberal model, an economic, political and social model that prioritizes the exploitation of resources and consumption. This model justifies “that agreements can be generated between families and political conglomerates that have created laws in favor of extractivism” (Participant Chile 3). Therefore, the participants identified that at the regulatory level, extraction is allowed over marine protection. This would promote overexploitation, which entails an excessive and indiscriminate exploitation of marine resources by the industries. According to some participants, especially Chileans, companies engage in this “to generate more income, to make fishing more profitable. This is a problem because in reality they do not allow the maintenance of the sea’s life cycle because they are interfering more than they should” (Participant Chile 17).

It’s been identified that, regarding the purpose associated with the sea, Peruvian and Chilean participants tend to focus on the recreational and economic use, respectively. While Peruvians link the sea to social activities and aesthetics more often, Chileans highlight that the sea is mainly seen by their society as a source for economic benefit, even above its protection. In those lines, Peruvian participants attribute the lack of concern about marine protection to a representation of the sea that solely focuses on the positive emotions generated by its recreational purpose, whereas Chilean participants relate the diminished marine protection to an economic and social model that allows harming extractive practices in search of economic gain.

### 3.2 Marine plastic pollution: socio-individual and structural causes and consequences

The prioritization of the recreational and economic use of the sea above its protection might be linked to marine pollution, which for the participants represents the main marine environmental problem. Marine pollution is seen as a set of various elements: “solid waste dumped by boats or people, or perhaps because there is a sewer or a drainage system nearby. A person can be swimming or enjoying themselves, and suddenly find garbage, excrement, dead birds, plastic” (Participant Peru 2).

In this sense, several types of marine pollution were identified by Peruvian and Chilean participants: (1) marine plastic pollution, the presence of plastic and microplastic in this environment, (2) pollution by other materials, such as glass, debris, animal remains and drainage, (3) oil spill pollution or other types of fuels, and (4) pollution from construction near the sea that causes its deterioration and produces waste that ends up in the beach. Among all these types, marine plastic pollution is recognized by the participants of both countries as the most visible and relevant problem that the sea faces: “the first thing that comes to my mind is plastic pollution, and how it damages life, the life of the animals that live there. For me, it is the most serious problem since I have seen it the most” (Participant Peru 11). Similarly, some participants report that the main pollution comes from the disintegration of plastic waste into microplastics in the sea or on the beach:

“Whenever I walk along the shore I see plastic, bottles, small bottle caps… beach toys and, especially, microplastics, which are those tiny plastics that are almost the size of a grain of sand. I can see them and you can spot a lot of them on the shore” (Participant Peru 4).

It has been identified, based on the participant’s perspective, that marine plastic pollution has socio-individual and structural causes. Within the socio-individual causes, the one most identified by the participants from both countries is the lack of knowledge, described as the limited information people have about marine plastic pollution and its consequences: “it has to do with the individual agency of people or small groups who are not aware of the effect that throwing plastic waste into the sea can have. Every little thing can increase the problem. They do not know the consequences” (Participant Peru 8).

Although, for some participants from Peru and Chile, scarcity of information is not the main cause of the problem, but the lack of responsibility towards marine protection. This means not taking responsibility for the behavior that pollutes the sea, even if they are aware of the damage it generates: “it’s not that you do not know you have to clean up litter or that there are not enough garbage cans, and even if there are no garbage cans you can take it with you and then throw it away, but people are irresponsible and lazy” (Participant Peru 16). This lack of responsibility, according to some participants from both countries, might stem from not associating their actions with the effects of marine plastic pollution: “there is no awareness that your action has repercussions, and if there is no awareness, you cannot grasp the logical connection; and since you do not see it and everyone else pollutes, you do not feel responsible” (Participant Peru 11). It also, based on the opinions of mostly Peruvian participants, stems from selfishness as it leads people to prioritize themselves and their needs over marine protection: “people think: ‘since I’m going to get out and I’m not going to come back, I can throw things here’ [the sea], so they pollute. This is what we see happening the most. And because they think that it is not their habitat or that they are not going to come back, they pollute it and do not worry about it” (Participant Peru 6).

These individual causes might be related to one of the most reported, mainly by Chileans, structural causes that involve societal and systemic factors that allow marine plastic pollution: the lack of environmental education, which refers to the lack of visibilization and broadcasting of information about environmental protection and the scarce active participation in environmental initiatives. According to some participants, the lack of environmental education hinders the perception of the sea as a crucial ecosystem for humanity that needs protection: “if they told us in school that the sea is important. Nobody talks about these things; it is very invisible. If they would talk about it, it would be different, it would be part of us” (Participant Chile 19).

Another structural cause of marine plastic pollution, identified by some participants from both countries, is the massive consumption of plastic, the widespread use of plastic due to its easy access and production that ends up reaching the sea: “it is easy to acquire it and, therefore, there is also mass consumption. People tend to pack and carry their food in plastic when they go to public spaces such as the beach” (Participant Peru 9). It should be noted that the lack of environmental education and the massive consumption of plastic are mainly attributed to users of the sea such as bathers and athletes, as well as coastal communities.

In this regard, some Peruvian and Chilean participants recognize that another structural cause of the problem is poor waste management, meaning the deficient management of waste systems in both countries, which begins with the way people dispose of it and ends with how it’s processed. The poor waste management, according to participants, can be attributed to a deficient handling of the problem by the authorities and companies: “at a systemic level, recycling is wrongly planned. It has been proven that, even if there are waste segregation containers, litter ends up all together in a sanitary landfill” (Participant Chile 9).

Based on the above, it has been identified that both socio-individual and structural causes are perpetuated over time due to a lack of regulation of plastic fabrication and consumption by the authorities, that consists of poor control and lack of sanctions established for individual, groups or companies’ polluting behavior. In that sense, some participants hold their governments responsible for allowing the perpetuation of marine pollution: “in large industries, if control measures or rules are not set, they will never be aware of the issue. It also applies to people. If the issue is not taken seriously by authorities by setting rules against marine pollution, people will continue doing it. This also replicates with the fisherman, if the authorities do not tell them anything and allow them to do their fishing activities without conscience or care, the problems will continue” (Participant Peru 19).

The perpetuation of marine plastic pollution, based on the opinions of Peruvian and Chilean participants, has an ecological, social and individual impact. At the ecological level, it is recognized that plastic causes severe damage to nature, manifested in the ecosystem disruption and the impact on climate change. In one hand, the disruption of the ecosystem includes the negative modification of the interaction between marine species and their environment, due to the presence of plastic waste: “the alteration of the habitat of the beach, of the water, of the species that live there… it has been seen that where there used to be nesting birds, today due to plastic pollution, they no longer remain in that area” (Participant Chile 15). On the other hand, plastic pollution is thought to be a catalyst for an environmental crisis that might have an impact on climate change: “I imagine that it is associated with the melting of the poles, the deterioration of the ozone layer, I think that marine pollution increases the problem” (Participant Peru 1).

At the societal level, the reported consequences of marine plastic pollution are the impact on the food chain, the scarcity of resources, and the difficulty in cleaning up the sea. According to the participants, the most relevant social impact would be the effects on the food chain, such as the ingestion of plastic waste by marine species that end up reaching humans through the food chain: “there are several studies that show that a great number of fish that we consume in the capital, that come from the sea, consume microplastics and therefore, when we eat them, we also ingest these microplastics” (Participant Peru 2). This effect is related to resource scarcity, the risk that the resources provided by the sea will be depleted due to the presence of plastic in large quantities: “yes, because it affects all of us. The animals begin to die, we run out of fishery resources” (Participant Peru 19). In this way, for some participants, this permanent presence of plastic waste through microplastics generates a difficulty in cleaning up the sea, and thus maintains the risks to the health and the future of societies.

At the individual level, most Peruvian participants report that marine plastic pollution results in a loss of enjoyment of the sea, understood as the low satisfaction in its recreational use due to the presence of plastic waste: “for athletes, it is not pleasant to enjoy a sea full of plastic” (Participant Peru 13). Since the recreational use of the sea is seen as one of its main purposes, the lack of it might trigger negative emotions in some of the participants, making them more aware of other negative effects of marine plastic pollution.

In fact, most participants have reported experiencing emotions such as anger, sadness and hopelessness. Anger refers to the feeling of being upset about marine plastic pollution: “sometimes it makes me angry, because I see it as something unfair, as something that should not be” (Participant Peru 11). On the other hand, sadness refers to the feeling of sorrow generated by seeing how pollution affects marine life: “sadness because due to us, animals that do not know how to defend themselves are directly affected” (Participant Chile 16). Participants also experience hopelessness because they perceive that other people, institutions and their authorities do not and will not take responsibility for dealing with the problem: “Sometimes the problem is huge for one person or a specific group to take charge. And one feels impotence to see how different companies that are mainly responsible for this pollution, due to plastic production, do nothing. I think: what am I going to do? what can I do?” (Participant Chile 4).

This hopelessness is associated with the participants’ assessment of the actions they take in response to marine pollution. Most of them perceive that they have limited self-efficacy in response to the problem: “more than anger, this situation makes me feel sorrow, and I try to do something about it. Yes, we try to educate as much as we can, but we cannot control everything, we cannot solve everything” (Participant Peru 21). In this sense, especially for Peruvian participants, there is a concern for future generations, understood as the concern produced by the belief that other generations will not be able to benefit from the sea due to pollution: “we can survive thanks to the sea and future generations also will. One time I heard: “I hope climate change… will be in 100 years,” but in 100 years other people will be alive. There will be people who will not be able to have these moments of enjoying the sea” (Participant Peru 11).

However, there is some hope left as a group of participants report feeling a need to protect the sea, which refers to the communities’ will to take actions to address marine plastic pollution and take care of the sea: “more than emotions, it’s the need to protect. That feeling that made me feel annoyed with the fact that it was dirty, untidy, we turned it into something positive. In the end it was like a manifestation of unification, we started to protect” (Participant Chile 3). The willingness to turn negative emotions towards marine plastic pollution into collective efforts for marine protection, although reported by a reduced number of participants, might be a useful path to promote pro-environmental behaviors.

As presented above, Peruvian and Chilean participants have a general knowledge of the causes and consequences of the problem that, although is superficial, reflects a general scope of individual and societal factors and stakeholders at play in perpetuating marine plastic pollution. This might be influenced by their college education; however no causal association can be made. It is relevant to highlight that Peruvian participants tend to focus more on individual causes and consequences rather than structural ones, in comparison to their Chilean counterparts. This pattern seems to repeat in their proposals for solution alternatives.

### 3.3 Individual and structural solution initiatives for marine plastic pollution

Finally, the participants suggested different individual and structural initiatives that they have or intend to have carried out or think that might be implemented by various stakeholders to reduce marine plastic pollution (see Figure 2). Regarding individual initiatives, the majority of participants from both countries recognize the importance of reducing plastic consumption, such as avoiding the use of plastic in daily life and switching to biodegradable products. Thus, they believe that “we can go shopping with our reusable cloth bags, so that people could appreciate its importance, I mean, there are people doing it, and it is not that complicated” (Participant Peru 12). This initiative could be useful to reduce the massive consumption of plastics, but it is stressed that to achieve this, the authorities and companies need to get involved in offering sustainable alternatives.

Another alternative that could be implemented with the collaboration of these stakeholders is beach cleanup, understood as picking up litter from the beach. Participants from both countries consider this as a useful initiative since it contributes, slowly, to the reduction of marine plastic pollution: “yes, it is slow, but effective. Although, with the support of some organization or the government, it could be more effective. People do it purely for the environment” (Participant Chile 18). Despite the perceived contribution of this action, some people, specifically Peruvians, consider it ineffective in the grand scheme of the problem, as it does not address the causes of marine plastic pollution: “it is not targeting the root cause of the problem itself, which is how plastic enters the sea not the cleaning of it” (Participant Peru 8). However, the general consensus is that beach cleanups might increase awareness about the relevance of marine protection.

Although most of the participants expressed not having been engaged in these initiatives, some of them, mostly Peruvian participants, report doing the following pro-environmental behaviors: picking up litter, understood as cleaning up their own or others’ waste when being on the beach, and calling out other people for disposing their waste on it by disapproving their behavior and sharing information about the consequences of the problem: “we keep a close eye on the beach goers to see if they litter… If they try to hide the litter, we intervene to guarantee the correct waste disposal, requesting them to ‘take their litter home, as they came here without it, and found the beach litter-free. Let us leave it as we found it’” (Participant Peru, 14).

Nonetheless, most participants recognize that their reported-proenvironmental behaviors are few and far between, thus they manifest having intentions to participate in more meaningful pro-environmental behaviors, such as recycling. Recycling involves collecting, sorting, and classifying plastics so it can be reused or taken to recycling centers to be managed or transformed. For those rare participants that do it, recycling might be a manifestation of a society’s increased concern for the environment: “at my house, we all recycle. Once my car is full, I take it to the recycling center. It’s satisfying to see the center’s containers filled with recyclable materials. This indicates the occurrence of a cultural shift” (Participant Chile 8). In that sense, for particularly Chileans, the purpose of the individual initiatives is to make marine plastic pollution more visible. This involves educating themselves about the issue, but also sharing it with others, raising awareness about it: “we must take action, protect our beaches and seas, educate those with little awareness, speak out when necessary, and actively participate in this process” (Participant Chile 16).

These individual level solutions must be supported by structural proposals with political impact. In this regard, for participants from both countries, a key initiative to take is environmental education, focused on promoting and broadcasting information about environmental protection with the involvement of the government and the citizens, in order to mitigate the lack of knowledge and the lack of responsibility towards the problem: “If in my community, I learn that plastic is harmful to the planet, I will refuse to use it, and if I see it I will try to recycle or reduce it. I will try to make a change, but if I have not been educated about plastic pollution, it would be hard for me not to do it” (Participant Chile 6).

In addition, participants propose structural initiatives that should be implemented as part of a collective involvement, understood as the joint participation of private companies, government institutions and citizens in generating pro-environmental significant change. In the case of companies, some participants, especially Chileans, consider that businesses should focus on reducing plastic manufacturing and replacing it with sustainable alternatives: “here in Chile there was also a bill in which supermarkets stopped giving out shopping plastic bags, so every time we went to the grocery store, we had to know that we should bring our reusable bag” (Participant Chile 4). Furthermore, some participants from both countries believe that companies should take actions and implement policies to reduce their environmental footprint.

Regarding the structural initiatives that should be taken by the government, Peruvian and Chilean participants mainly suggest the establishment of laws and sanctions. This implies the elaboration of norms that regulate and punish people’s polluting behavior. In other words, the authorities need to “enter a little more into politics, create new laws, establish regulation, and take strict measures to deal with cases of pollution” (Participant Peru 12). It is important to highlight that, for a small group of Chilean participants, the Constitutional Convention, the body in charge of drafting the new constitution in Chile, should support this initiative. Also, most of the Peruvian participants consider significant the involvement of the authorities in improving waste management. They believe that “those in the government have to make a good recycling plan, manage the waste, collect it from the whole district and city, gather it in a place where it will be buried, burned, and segregated between plastic and organic waste” (Participant Peru 18).

According to mostly Chilean participants, these pro-environmental initiatives have been undertaken by civil society rather than the government or private companies, thus they believe that this stakeholders need to finance and promote pro-environmental behaviors since its their responsibility to do so: “initiatives are achieved more at the local level than at the governmental level, there should be support for self-managed organizations from municipalities or the government because it is something they are supposed to do, but we do it for them” (Participant Chile 2).

Faced with a limited engagement of the authorities in addressing marine plastic pollution, some participants propose that citizens increase their political involvement, which entails a political participation in favor of environmental protection by selecting authorities who support it, and organizing the community for protest: “just as they call to protest for certain social issues, they should protest for environmental issues. We and those to come are the ones who will be the counties’ leaders” (Participant Chile 19). Along with this political involvement, some participants, specifically Chileans, believe that academic research should be encouraged, which implies carrying out research with the purpose of learning and informing citizens and other relevant social stakeholders about the causes and consequences of the problem in order to develop possible solutions.

In those lines, some participants from Peru and Chile recognize that the main purpose of pro-environmental initiatives should be the revalorization of the sea, which entails the acknowledgement of its intrinsic value and the prioritization of marine protection at the societal and governmental level. For this, a joint effort is needed: “on one hand, individuals need to stop polluting marine areas, on the other hand, raising awareness within large companies is necessary to make them understand the significance of the sea; generating a collective environmental concern that can transcend in time” (Participant Peru 5). Therefore, although Chileans participants tend to focus more on the role that the government and private companies have in establishing structural initiatives than the Peruvian participants who mainly highlight the individuals’ responsibility, there is a general consensus that to effectively address marine plastic pollution various stakeholders must work integrally in generating, institutionalizing and participating in individual and structural initiatives that pave the way for a cultural change towards marine protection.

## 4 Discussion

Peruvian and Chilean citizens represent the sea as a space that provides refuge, disconnection from routine and, overall, connection with nature. This is based on affective experiences associated with being close to the sea (Perkins, 2010), such as tranquility and happiness. In this way, the sea acquires positive value from the meaning that the participants build around it (Valera et al., 2006).

### 4.1 An anthropocentric and neoliberal view of the sea

According to some studies, having a connection with nature might generate a greater environmental concern (Schultz, 2000) and a commitment to protect the environment (Wu and Zhu, 2021). However, in line with studies that found a gap between pro-environmental attitudes and pro-environmental behavior (Newsome and Alavosius, 2011; Páramo, 2017; Olivera et al., 2020), our results suggest that although the positive perceptions of the sea might encourage intentions of engaging in pro-environmental behaviors, in the case of most participants these intentions do not translate into pro-environmental behaviors, especially if it involves greater effort. Participants acknowledge that even if they feel connected to the sea, they do not engage in collective actions aimed at reducing marine plastic pollution in a systematic way. Even those who report having engaged in pro-environmental behaviors refer to an individual level of action, such as picking up litter from the beach. This distance between the participant’s environmental concern and their behavior could be due to the fact that their positive perception of the sea is more linked to the recreational benefits it brings them rather than to a commitment to its protection regardless of the costs or benefits.

The recreational use, in addition to fulfilling the cultural function of providing a space for aesthetic manifestations and spiritual well-being (Intergovernmental Panel on Climate Change (IPCC), 2014; Thushari and Senevirathna, 2020), allows enjoyment through leisure and social interaction. The prioritization of this recreational use of the sea, according to the participants, leads people to not care about it beyond the experiences it provides them. In this case, the concern about plastic pollution could be linked to selfish values, which influence their decision to engage in pro-environmental behaviors according to the cost or benefit related to it (Steg and de Groot, 2012). Therefore, people tend to engage in pro-environmental behaviors that represent less costs from them, such as occasionally picking up litter and calling out pollution behavior rather than reducing the use of plastic or recycling, while still benefiting from their connection to the sea.

The economic use attributed to the sea fulfills an extractive function, as this ecosystem is seen as a supply of food and other natural resources (Intergovernmental Panel on Climate Change (IPCC), 2014; Saavedra and Mardones, 2021). Participants from both countries report that, as a result of this attribution, overexploitation represents one of the main problems that damages the sea and has repercussions on its pollution. This perception of the sea as a vehicle for extractive and economic profit is associated with the neoliberal model, defined by the participants, especially Chileans, as the economic, political and social model that prioritizes the exploitation of resources and consumption.

The neoliberal model would be evidenced in the decision of the authorities of both countries to apply laws on the manufacture and use of single-use plastics to specific locations, such as tourist areas, so that they do not generate changes to business sectors of greater economic interest for the country, such as small and medium companies in Peru (Morales, 2022), and aquaculture industries in Chile (Kiessling et al., 2017). The passing of the law in tourist locations might respond to the fact that tourism in coastal regions is an economic activity that relies on the recreational use of the sea, which is harmed by plastic pollution in it; thus, in the authority’s perspective, marine protection in coastal regions would be aligned with the economic interests. Conversely, laws that prioritize marine protection over profit in regions whose industries focus on the exploitation of marine resources might be perceived by the authorities as threats to the economic interests of the population, even though different civil society organizations and, in the case of Chile, local authorities have been making efforts to bring to their attention the consequences of plastic pollution (Oceana Peru, n.d.; Sierra, 2018b; Cristi et al., 2020). A similar situation has been found in contexts whose economic model is neoliberal: the proposed solutions to the environmental crisis are rejected, as they challenge the neoliberal system that responds to social sectors that seek the greatest possible profit, even if this results in the government’s inability to address environmental issues (Geiger et al., 2022).

Hence, the decision of authorities to apply pro-environmental policies only in sectors where this would be beneficial to their economy could be influenced more by altruistic values than by biospheric values, given that the main interest is in protecting the economic welfare of the population and not in marine conservation (Steg and de Groot, 2012). In that line, we propose that the altruistic values that might be behind the authority’s decisions correspond to the preservation of the economic interests of today’s pollution rather than concern for future generations. The lack of addressing marine plastic pollution consequences on a larger scale by the authorities might be the reason why a few of the participant’s report being concerned for future generations, and why these reports have come from the Peruvian sample, since Peru, in comparison to Chile, has been less in favor of prohibiting single-use plastics (World Wide Fund for Nature, 2022).

Therefore, the environmental concern about marine plastic pollution corresponds more to, what the New Ecological Paradigm calls, the anthropocentric perspective, which is focused on the superiority of human beings over nature. This perspective directs environmental concern towards the consequences that the environmental damage has for the individual or for society (Dunlap et al., 2000). Thus, it was found that the concern of the participants falls more on the social and individual consequences of the problem, such as the loss of enjoyment of the sea or the impact on the food chain, than on the ecological ones, like the disruption of the ecosystems.

Under that anthropocentric and neoliberal perception of the sea, the environmental attitude is characterized in this study, at a cognitive level, by the lack of information about the problem, its consequences and its relationship with human behavior. At the affective level, marine plastic pollution causes negative emotions, such as sadness and annoyance, next to hopelessness, which is triggered by the acknowledgment of the magnitude of the problem and the negative environmental attitude of other citizens. At the dispositional level, participants recognize that their societies have a low predisposition to marine protection, manifested in the high consumption of plastic, due to its easy manufacture, use and disposal (Schröder et al., 2020), in the poor waste management, and in the lack of oversight and establishment of plastic legislation to regulate the industries’ bad practices (Vergara-Schmalbach et al., 2014).

In this context, both Peruvians and Chileans present limited individual efficacy of response, i.e., they perceive that their pro-environmental behaviors are insufficient to solve the problem. The latter would explain why the participants, despite having a connection with the sea, do not consistently maintain a commitment to protect this environment, limiting their actions to an individual, loosely organized and low-impact level (Hoffman and Graham, 2015). This limited individual efficacy of response could be understood by the proposed model of socialization of pro-environmental behavior (see Figure 1), which depends on the interaction and interdependence between the different systems in which a person develops. Thus, the individual pro-environmental behaviors that the person carries out in the different systems to which they belong (microsystem) must be integrated with municipal or national actions (exosystem) and must be supported by values of marine protection and conservation from the society to which they belong (macrosystem) in order to achieve the desired impact.

This environmental attitude is linked by the participants to two causes of the problem: selfishness and overexploitation. These causes are part of the macrosystem, that is, the system of beliefs and values of the societies to which the participants belong and which influence individual and institutional behavior. In the Peruvian case, marine plastic pollution is attributed to individual characteristics such as selfishness, which is usually related to individualism. The latter is seen negatively in collectivist societies such as the Peruvian one, as it prioritizes individual development and well-being (Yamamoto and Feijoo, 2007) over the collective ones. The Peruvian participants identify this prioritization of individual well-being over collective well-being as selfish acts of people in the face of the problem. In the Chilean case, the participants recognize a link between the overexploitation of the sea and the neoliberal model, as they report that industrial fishing generates large quantities of plastic waste into the sea. They also point out the prioritization of the consumption of plastic products over marine protection. It is important to note that both causes are interrelated and are part of the neoliberal model to which both societies belong. Peruvian participants tend to identify selfishness as a trait of their society, while Chilean participants tend to link overexploitation to the country’s economic system. This more critical and structural view of the problem in the Chilean participants could be related to the political landscape that Chile is going through and to the more active political involvement of its citizens (Vera, 2017; Allain, 2019).

Neoliberalism as a social and economic system has disarticulated the relationship between government and civil society (Rottenbacher and Schmitz, 2012). In Peru, over the last decades neoliberalism brought economic growth at the expense of increasing informality, inequality and institutional distrust, which caused civil society to disengage from its political role and to not question the economic and political system (Vergara, 2020). In Chilean, although the neoliberal economic system has been questioned, there is still no commitment to change due to the fact that Chilean society maintains its tendency to legitimize social hierarchies (Valencia-Moya, 2018). This social dominance orientation has been found in other studies to be negatively related to pro-environmental attitudes and environmental policies (Häkkinen and Akrami, 2014).

### 4.2 What it’s being done: individual actions and local policies

**Figure 3:**
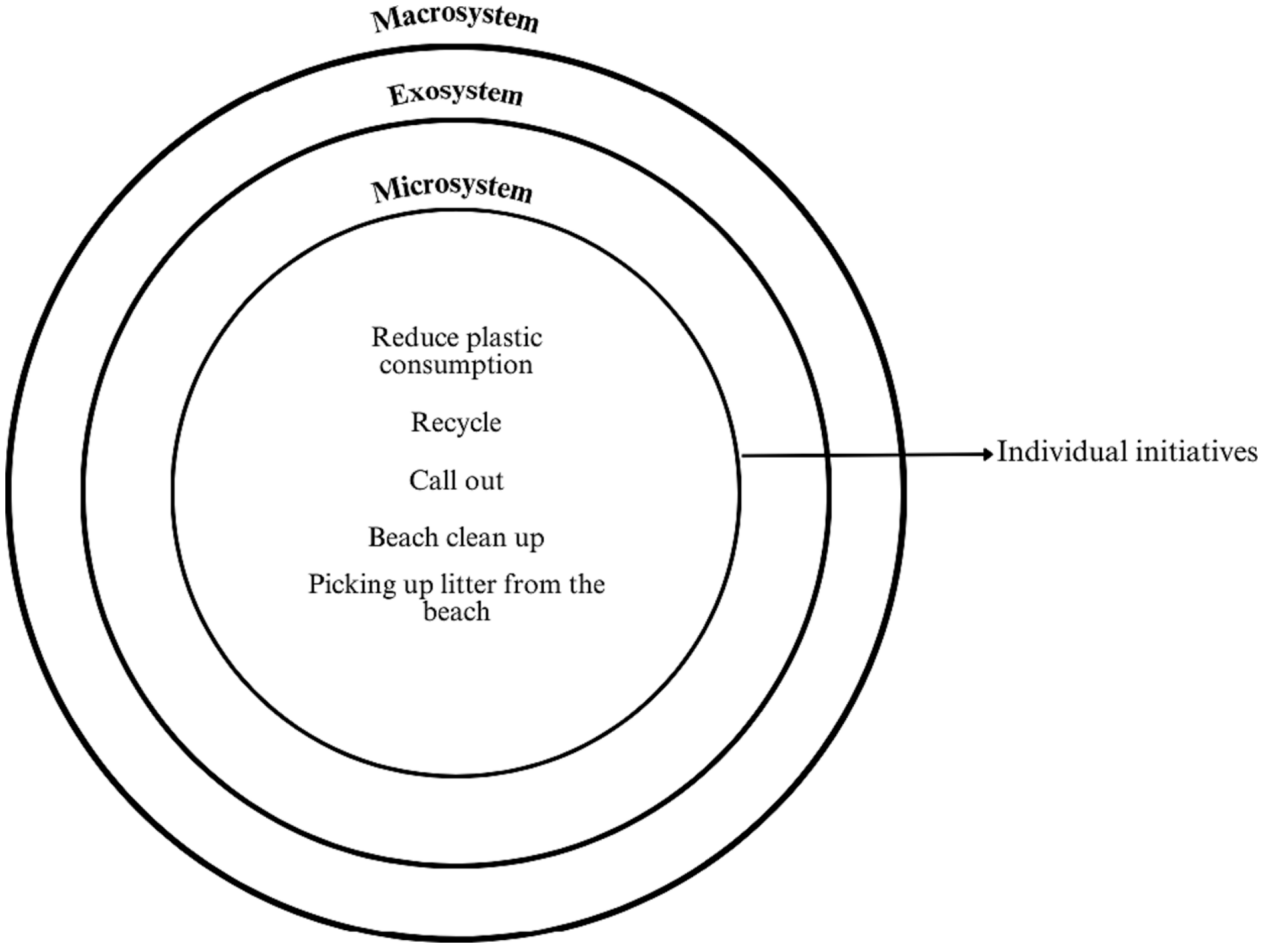
Participants’ perception of individual solution initiatives for marine plastic pollution. Elaborated by the research team.

Based on the above, two of the four levels of pro-environmental behavior are mainly identified (see Figure 3): individual actions and local level policies (Hoffman and Graham, 2015). Individual actions are reduced to beach clean ups, picking up litter and reprimanding polluting behaviors, which reflects a general and limited view at possible solutions to address the problem, since, instead of addressing the causes, they are aimed at mitigating the effects. Despite this, for the participants, mainly Peruvians, such actions are feasible to implement in practice and promote the visibility of the problem. It should be noted that this type of initiative is usually developed in immediate contexts where the individual belongs and participates actively, such as volunteer, family or friend groups, which correspond to the personal microsystem (Bronfenbrenner, 1979). In this sense, the attitudes towards marine plastic pollution held by systems closest to the individual influence their attitudes towards the problem, and vice versa.

Reducing plastic consumption and recycling are other individual initiatives aimed at mitigating plastic pollution and informing about its consequences, which, according to participants, require the collective involvement of citizens, businesses and government institutions. While participants report having the intention to engage in these individual actions, they recognize that the involvement of social stakeholders and institutions involved in policy making and with the power to take pro-environmental behaviors to a more institutional level is needed. This, according to Bronfenbrenner’s model, would correspond to the interaction between the behaviors involved in the mesosystem with the decisions, actions, policies or collaborations that can be made among various social stakeholders at the exosystem level. It is proposed that for individual actions to have an impact outside the individual, family or community sphere, it is necessary for various stakeholders and institutions to support, promote and replicate these actions through the implementation of local-level policies.

In this way, the reduction of plastic consumption and recycling, articulated with an improvement in waste management, would provide greater institutional support to the objective of reducing marine plastic pollution. Moreover, academic research, which can take place in the exosystem, would allow the elaboration of public policies based on scientific evidence, under which authorities could establish laws and sanctions regarding the problem. Although local policies would drive concrete actions, these represent a necessary step to achieve a profound change in society characterized by the establishment of a system of beliefs, policies and values focused on environmental protection (Hoffman and Graham, 2015). Even though, in Chile some initial steps have been taken to involve different stakeholders in addressing plastic pollution (Cristi et al., 2020), this would be difficult to develop and/or improve in both countries, since, according to the participants, there is a low commitment from the authorities to protect the environment at a more structural level.

### 4.3 What needs to be done: public policies and environmental belief system

**Figure 4:**
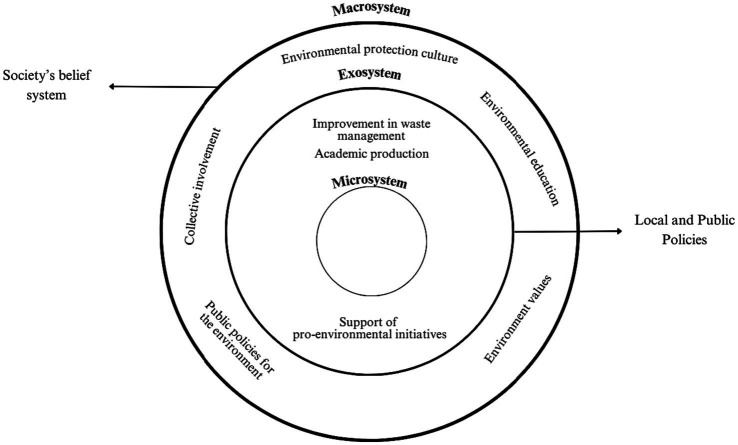
Participants’ perception of structural solution initiatives for marine plastic pollution. Elaborated by the research team.

As can be seen in Figure 3, from the point of view of the participants, the proposed solution initiatives at the individual level are inefficient for reducing marine plastic pollution, since they only influence the microsystem. In this sense, participants believe that ideally these actions should be complemented by structural initiatives that involve various stakeholders. Structural initiatives, seen in Figure 4, belong to the exosystem and macrosystem levels, in which a set of local and public policies together with a social belief system would reduce marine plastic pollution. It should be noted that the next two levels of pro-environmental behavior, a set of public policies promoted by interest groups and a culture of environmental protection (Hoffman and Graham, 2015), are less recognized to be executed in the participants’ perception. These structural levels of pro-environmental behavior play the role of sustaining individual actions and local policies (Bugallo, 2011).

An initiative known by the participants, especially Chileans, that corresponds to the public policy level is the support of pro-environmental initiatives. Although it is identified as a possible solution, most participants have little knowledge of initiatives focused on marine protection, such as “Hazla Por Tu Playa” (Peru) or “Científicos de la Basura” (Chile). Even though, specifically in Chile an informal alliance has been established between different stakeholders to take action against plastic pollution (Urbina et al., 2020), in the perspective of the participants of both countries there is a weak national presence of environmental activism articulated with governmental and non-governmental institutions. In fact, these initiatives tend to arise from the concern of citizens and not necessarily from the governments, which would demonstrate why the participants do not acknowledge the practical implementation of structural measures, even though they are considered important to solve marine plastic pollution.

According to Bronfenbrenner’s (1979) ecological model, this type of public initiatives would correspond to the exosystem, since they are spaces in which the individual does not necessarily have a direct participation, but indirectly influence their behavior. Thus, despite the fact that most of the participants are not actively involved in an environmental activist organization, the activities that these types of entities carry out in favor of marine conservation have repercussions, in some way, on the environmental actions of the individual. However, the latter would not be possible if there is not, in turn, a system of beliefs or values that promote environmental protection, which corresponds to what is known as the macrosystem (Bronfenbrenner, 1979). That is, the cultural elements of the macrosystem have influence on the lower order systems, so, as other studies have suggested (see Wichmann et al., 2022), if this system does not promote a cultural proposal that encompasses positive values and attitudes in favor of the environment, the development of pro-environmental behaviors at the other levels will hardly take place. This is because the macrosystem influences the way in which individuals behave within their social context (Bronfenbrenner, 1979).

However, it can be observed that there is not an acknowledgement of more wide-ranging levels of pro-environmental behavior, beyond individual actions and, to a lesser extent, policies at the local level. In that sense, there is also no acknowledgement of a belief system rooted in society that promotes environmental protection (Hoffman and Graham, 2015). On the contrary, it dominates a culture of exploitation, supported by a neoliberal system, which serves the economic interests of the authorities, private companies and plastic industries. In this context, a measure that, according to the participants, can confront this extractive predisposition and promote a culture of protection is environmental education, supported by the respective authorities and internalized by the general public.

Environmental education programs applied in Chile, with high school students, had a positive impact on the development of greater environmental awareness and environmental knowledge about the effects of debris in marine environments (Bravo et al., 2009). These interventions would not only increase positive environmental attitudes towards the sea, but also promote pro-environmental behaviors, such as improved plastic consumption practices (Jaime et al., 2023). In Peru, although there is no evidence of successful environmental education programs, awareness campaigns led by environmental activist organizations, such as “HAZla por tu Playa,” have promoted significant pro-environmental behaviors. This organization has managed to convene more than 10,000 volunteers to carry out more than 1,235 beach cleanup campaigns nationwide and more than 52 awareness-raising campaigns, increasing environmental awareness in the participants and encouraging them to improve their plastic consumption habits and veer into a sustainable one (SPDA Actualidad Ambiental, 2023).

Therefore, this study proposes that environmental education should include cognitive, affective and behavioral components that express a predisposition to act in favor of the environment. In this sense, it is necessary to generate greater knowledge in the population through the visualization of the causes and consequences of marine plastic pollution and the various strategies to address it (cognitive). Furthermore, to encourage environmental concern for the effects of plastic pollution on humans and nature, a connection with nature and a sense of responsibility towards the marine ecosystem must be nurtured (affective). Finally, it is crucial to supply sustainable alternatives to replace plastic products and promote effective waste management, such as recycling (behavioral). Integrally, this type of education should be transversal throughout the individual’s development, in order to promote values, beliefs, attitudes and practices focused on environmental protection. Thereby, environmental sustainability and social welfare might be achieved.

## 5 Conclusion

In synthesis, the environmental representation of the sea, characterized mainly by the attribution of recreational and economic purposes, orients environmental concern towards the consequences of marine plastic pollution on individuals and society. This would reflect, according to the New Environmental Paradigm (Dunlap et al., 2000), an anthropocentric perspective of the problem, since environmental concern about the sea would be based on the value it has for the maintenance and improvement of the quality of human life (Thompson and Barton, 1994). In this sense, instead of engaging in pro-environmental behaviors based on biospheric values that assign an intrinsic worth to nature, the various social stakeholders will behave according to selfish values, which emphasize the cost and benefit for themselves, or altruistic values, which prioritize, in this case, the economic interests of society.

Furthermore, it is found that the participants’ pro-environmental behaviors correspond to individual actions, developed in their personal microsystems, whose objective is to generate visibility and awareness of the problem in the closest social groups. It is recognized that for these actions to have an impact that transcends family, work or friendship contexts, a collective involvement with companies, institutions and academia is needed to take these pro-environmental behaviors to a structural level. In this sense, the pro-environmental behaviors carried out individually or collectively, corresponding to the microsystem and mesosystem, should be articulated with local, national and international policies established, promoted and executed by various social stakeholders such as companies, governmental and non-governmental organizations present in the exosystem. However, this articulation between individual actions and public policies might be hindered by the neoliberal system which promotes a culture of exploitation of the sea, attributable to selfishness, in the Peruvian case, and overexploitation, in the Chilean case.

Thus neoliberalism, as a social and economic system that prioritizes economic gain over the environment and obstructs pro-environmental behaviors (Geiger et al., 2022), might be a core factor in the perpetuation of marine plastic pollution. Although both countries have adopted this system and currently deal with its social consequences, it’s relevant to highlight that only the Chilean participants verbally acknowledge its possible influence on the causes of marine plastic pollution and the authorities’ lack of commitment towards resolving it.

Therefore, there is a need to build and promote a culture of marine protection that encourages citizens, academia, industries and authorities to adopt more environmentally sustainable forms of consumption and production, in order to reduce the use of plastics and, ultimately, plastic pollution. To achieve this, starting from the anthropocentrism that prevails in society, it is suggested to use selfish values, linked to leisure and resource extraction, to promote marine protection in order to preserve its recreational and economic use. Upon this, it is proposed to promote altruistic values that re-orient marine protection in favor of the maintenance and improvement of the quality of life of the individual and society. This to, finally, develop biospheric values under which harmony is sought between the welfare of society and environmental protection, so that environmental concern is oriented towards the intrinsic value of nature, beyond any anthropocentric interest.

In oversight, this study contributes to literature by integrating two theoretical models, Hofmann and Graham’s levels of analysis of pro-environmental behavior and Brofrenbrenner’s model of socialization and assessing the role of each level of analysis into the systems of socialization to analyze the environmental attitudes and behaviors of Peruvian and Chilean participants, in light of possible influences of political, social and economic factors, like the neoliberalism system.

Regarding the limitations of the study, it should be noted that participants were mostly urban residents, which hinders the opportunity to assess if the proximity to the sea has any influence on the connection with nature or positive perceptions of the sea. Likewise, another limiting factor is that during the analysis of the results no differences were found based on demographics, since most of the participants shared similar social, economic and education backgrounds. In addition, the qualitative study design did not allow further generalizations of the findings.

Lastly, it is recommended to carry out research with strategic social stakeholders involved in decision making, such as authorities and entrepreneurs in the plastic industry. In addition, research should be expanded to explore the impact that plastic consumption habits have on marine pollution.

## Data availability statement

The original contributions presented in the study are included in the article/Supplementary material, further inquiries can be directed to the corresponding author.

## Ethics statement

Ethical review and approval was not required for this study involving human participants in accordance with the national legislation and institutional requirements of Pontificia Universidad Católica del Perú, institution responsible for this project. The studies were conducted in accordance with the corresponding local legislation and institutional requirements. Written informed consent for participation was not required from the participants or the participants’ legal guardians/next of kin in accordance with the corresponding national legislation and institutional requirements.

## Author contributions

FS: Conceptualization, Data curation, Formal analysis, Investigation, Methodology, Writing – original draft, Writing – review & editing. MM: Conceptualization, Data curation, Formal analysis, Investigation, Methodology, Writing – original draft, Writing – review & editing. ST: Conceptualization, Data curation, Formal analysis, Investigation, Methodology, Writing – original draft, Writing – review & editing. MT: Funding acquisition, Investigation, Methodology, Supervision, Writing – review & editing. JB: Investigation, Methodology, Resources, Writing – review & editing. AE: Conceptualization, Formal analysis, Funding acquisition, Investigation, Methodology, Project administration, Supervision, Validation, Writing – original draft, Writing – review & editing.
