# Supplementary material for: Sea of plastic: representations of the sea and pro-environmental attitudes and behaviors regarding marine plastic pollution in Peru and Chile
Source: Front Psychol. 2024 Jan 18;14:1308796. doi: 10.3389/fpsyg.2023.1308796 (PMC10832806; doi:10.3389/fpsyg.2023.1308796)
Supplement: Supplementary file 1 [file Data_Sheet_1.docx]

***Supplementary Material***

**Geographical map**


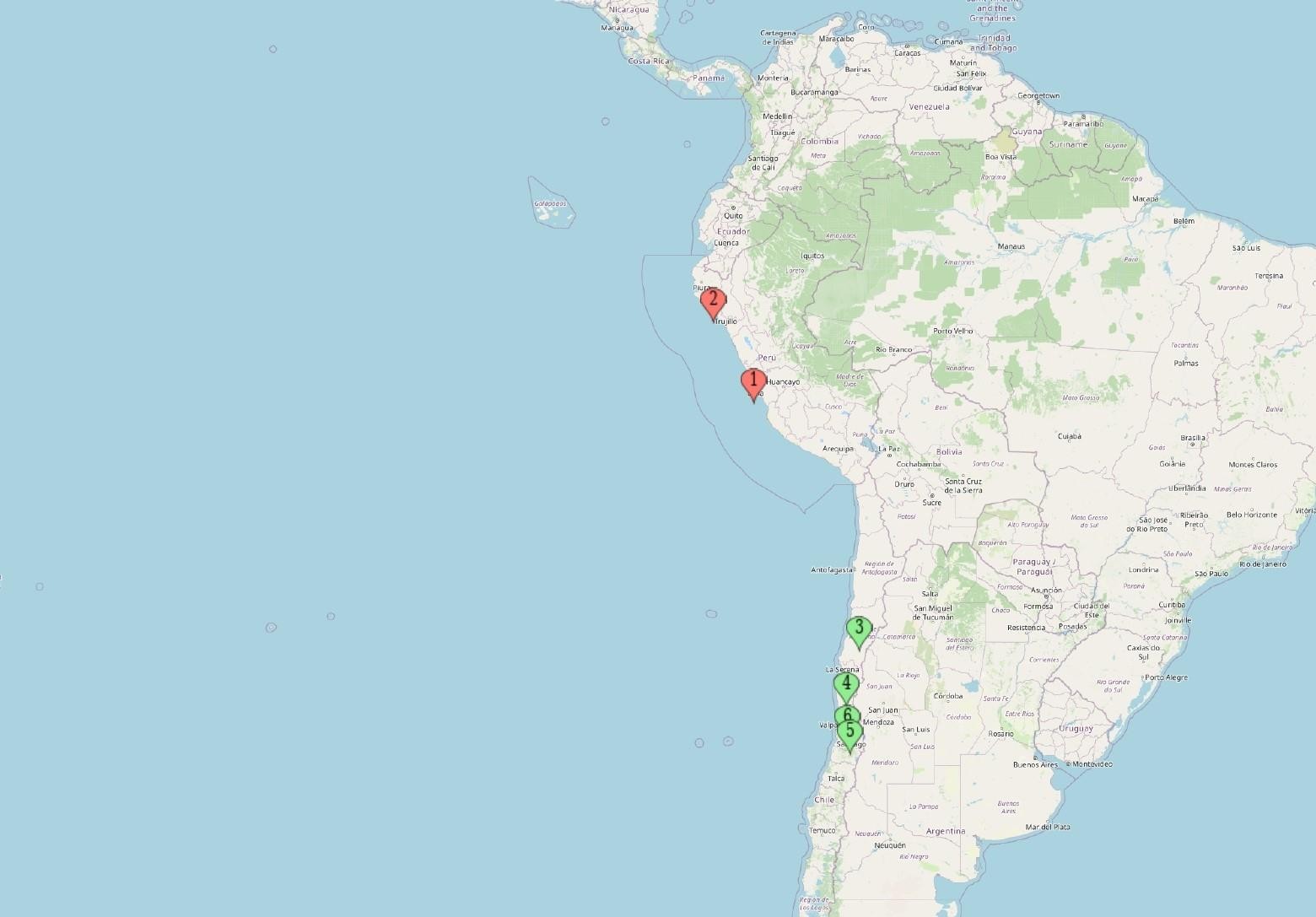


*Note:* Perú (1. Región de Lima Metropolitana, 2. Región de Lambayeque); Chile (3. Región de Atacama, 4. Región de Coquimbo, 5. Región Metropolitana de Santiago, 6. Región de Valparaíso).

**Participants’ socio-demographic data**

| **N. º** | **Country** | **Sex** | **Age** | **Educational level** | **Activity** | **Activity frequency** |
| --- | --- | --- | --- | --- | --- | --- |
| 1 | Peru | F | 36 | Higher education | User | Every weekend |
| 2 | Peru | M | 25 | Higher education | Sportsperson | Once a week |
| 3 | Peru | F | 27 | Higher education | User | Summer |
| 4 | Peru | F | 24 | Higher education | User | Summer |
| 5 | Peru | M | 19 | Higher education | Sportsperson | Three times per week |
| 6 | Peru | F | 22 | Higher education | Sportsperson | Once a week |
| 7 | Peru | F | 25 | Higher education | User | Summer |
| 8 | Peru | M | 26 | Higher education | No User | Once every two months |
| 9 | Peru | F | 32 | Higher education | No User | Twice a year |
| 10 | Peru | F | 18 | Higher education | No User | Once a year |
| 11 | Peru | F | 22 | Higher education | No User | Twice a year |
| 12 | Peru | M | 23 | Higher education | Sportsperson | Twice a month |
| 13 | Peru | M | 28 | Higher education | Sportsperson | Twice a month |
| 14 | Peru | M | 33 | Higher education | Sportsperson | Once a week |
| 15 | Peru | M | 20 | Higher education | Sportsperson | Once a month |
| 16 | Peru | F | 23 | Higher education | User | Twice a week |
| 17 | Peru | M | 23 | Higher education | User | Once a month |
| 18 | Peru | M | 54 | Higher education | User | Summer |
| 19 | Peru | F | 24 | Higher education | Sportsperson | Three times per week |
| 20 | Peru | F | 23 | Higher education | User | Summer |
| 21 | Peru | F | 23 | Higher education | Sportsperson | Once to twice a week |
| 22 | Peru | F | 27 | Higher education | User | Four times a year |
| 23 | Peru | F | 40 | Higher education | User | Every day |
| 24 | Peru | F | 33 | Higher education | User | Once a month |
| 25 | Chile | F | 22 | Higher education | Sportsperson | Twice a week |
| 26 | Chile | F | 39 | Higher education | User | Once a week |
| 27 | Chile | F | 32 | Vocational education | User | Once a month |
| 28 | Chile | F | 24 | Higher education | User | Once a month |
| 29 | Chile | F | 26 | Higher education | User | 6 times a year |
| 30 | Chile | F | 41 | Higher education | User | Twice a week |
| 31 | Chile | F | 40 | Higher education | Sportsperson | Every day |
| 32 | Chile | F | 40 | Higher education | User | Twice a week |
| 33 | Chile | F | 26 | Higher education | Sportsperson | Once a week |
| 34 | Chile | M | 22 | Higher education | Sportsperson | Once a week |
| 35 | Chile | F | 46 | Higher education | User | Twice a week |
| 36 | Chile | F | 25 | Higher education | User | Once a month |
| 37 | Chile | F | 60 | Higher education | User | Every day |
| 38 | Chile | F | 49 | Higher education | User | Every day |
| 39 | Chile | F | 28 | Higher education | User | Once a month |
| 40 | Chile | F | 34 | Higher education | User | Twice a week |
| 41 | Chile | M | 28 | Higher education | User | Every day |
| 42 | Chile | M | 25 | Higher education | User | Summer |
| 43 | Chile | M | 35 | High school | User | Summer |
| 44 | Chile | M | 34 | Vocational education | User | Twice a year |

**Codebook**

| **Family Code** | **Code** | **Code Definition** | **Frequency by quote** | **Frequency by participant** | **Peru** | **Chile** |
| --- | --- | --- | --- | --- | --- | --- |
| Sea Representations | Magnificence | The feeling of being in front of an immense element | 27 | 15 | 7 | 8 |
| Sea Representations | Unknown | The feeling of not knowing or controlling the sea | 16 | 11 | 3 | 8 |
| Sea Representations | Dangerous | The sea as a potentially dangerous place, thus people must be careful and be respectful | 13 | 10 | 3 | 7 |
| Sea Representations | Freedom | Feeling of not having restrictions or responsibilities | 10 | 3 | 1 | 2 |
| Sea Representations- Positive | Origin of life | The sea is where life was created, where biodiversity is found | 36 | 25 | 10 | 15 |
| Sea Representations- Positive | Disconnection from routine | A space to distance yourself from work, associated with freedom | 19 | 15 | 9 | 6 |
| Sea Representations- Positive | Refuge | The sea as a safe space, appropriate for relaxation and expression of emotions | 23 | 14 | 8 | 6 |
| Sea Representations- Positive | Aesthetic | The sea as beautiful or aesthetically pleasing | 15 | 13 | 4 | 9 |
| Sea Representations- Positive | Resistance symbol | The sea represents a symbol that allows for the revalorization and resignification of indigenous identities | 4 | 1 | 0 | 1 |
| Sea Representations-Use | Recreative use | The sea as a space for recreative activity (play, sports, leisure, social interaction) | 53 | 27 | 19 | 8 |
| Sea Representations- Use | Economic use | The sea as a space for economic activity (resource extraction) | 39 | 19 | 9 | 10 |
| Sea Representations-Use | Cultural use | The sea as a valuable element for cultural resignification and revalorization, a space with history | 15 | 4 | 1 | 3 |
| Positive Feelings | Tranquility | The feeling of no having problems or worries | 53 | 36 | 21 | 15 |
| Positive Feelings | Connection with nature | The feeling of being connected to the sea and with the life that converges in it | 47 | 23 | 12 | 11 |
| Positive Feelings | Happiness | The feeling of wellbeing, exaltation and fun | 17 | 12 | 8 | 4 |
| Positive Feelings | Energy renewal | Renewal of positive energy, going out the sea with a feeling of wellbeing | 20 | 12 | 6 | 6 |
| Positive Feelings | Place attachment | The attachment between the person and the sea | 7 | 4 | 2 | 2 |
| Marine Pollution | Beach pollution | Pollution of the beach and/or the sea | 50 | 36 | 20 | 16 |
| Marine Pollution | Marine plastic pollution | Pollution by plastic objects and micro-plastics | 58 | 35 | 20 | 15 |
| Marine Pollution | Pollution by other materials | Pollution by glass, debris, animal, drainage residue | 34 | 23 | 12 | 11 |
| Marine Pollution | Oil spill Pollution | Pollution by petroleum, gasoline | 16 | 12 | 8 | 4 |
| Marine Pollution | Pollution from construction | Pollution caused by construction waste | 15 | 9 | 2 | 7 |
| Marine Plastic Pollution-Causes | Lack of knowledge | Lack of information about marine plastic pollution and its consequences | 44 | 27 | 12 | 15 |
| Marine Plastic Pollution-Causes | Lack of responsibility | Not taking accountability for marine plastic pollution | 52 | 24 | 12 | 12 |
| Marine Plastic Pollution-Causes | Lack of environmental education | Little environmental education and the scarce active participation in pro-environmental initiatives | 43 | 21 | 9 | 12 |
| Marine Plastic Pollution-Causes | Poor waste management | Poor waste management in a national level, this includes the lack of information about it | 31 | 20 | 11 | 9 |
| Marine Plastic Pollution-Causes | Selfishness | To consider only themselves and their priorities in a way that marine protection is an inconvenience | 31 | 19 | 14 | 5 |
| Marine Plastic Pollution-Causes | Lack of regulation | The lack of regulation and sanction of polluting behavior | 38 | 18 | 7 | 11 |
| Marine Plastic Pollution-Causes | Exploitation of the sea | The low valorization and prioritization of marine protection | 41 | 16 | 7 | 9 |
| Marine Plastic Pollution-Causes | Overexploitation | The exploitation of marine resources by companies | 28 | 14 | 3 | 11 |
| Marine Plastic Pollution-Causes | Massive plastic consumption | The generalized use of plastic because of its easy access and low cost | 27 | 13 | 8 | 5 |
| Marine Plastic Pollution-Causes | Neoliberalism | Economic, political and social model that prioritizes the exploitation of resources and consumption | 21 | 9 | 1 | 8 |
| Marine Plastic Pollution-Consequences | Ecosystem disruption | Negative disruption of the interaction between marine species and their environment | 67 | 34 | 18 | 16 |
| Marine Plastic Pollution-Consequences | Impact on the food chain | Plastic ingestion by marine fauna and humans due to the impact on the food chain | 49 | 33 | 19 | 14 |
| Marine Plastic Pollution-Consequences | Low enjoyment of the sea | Low satisfaction with the recreative use of the sea due to marine pollution | 38 | 23 | 17 | 6 |
| Marine Plastic Pollution-Consequences | Difficulty cleaning up | Difficulty in cleaning up the sea from micro-plastics | 17 | 13 | 9 | 4 |
| Marine Plastic Pollution-Consequences | Resource scarcity | The risk of running out of marine resources | 15 | 12 | 7 | 5 |
| Marine Plastic Pollution-Consequences | Impact on climate change | The repercussions of marine plastic pollution on climate change | 8 | 5 | 2 | 3 |
| Marine Plastic Pollution-Attitudes | Limited self-efficacy | The sense of efficacy of their actions in decreasing marine plastic pollution | 36 | 24 | 11 | 13 |
| Marine Plastic Pollution-Attitudes | Anger | The feeling of being upset about the causes and consequences of marine plastic pollution | 25 | 21 | 9 | 12 |
| Marine Plastic Pollution-Attitudes | Hopelessness | The belief that people will continue to produce marine pollution and nothing will change | 23 | 17 | 11 | 6 |
| Marine Plastic Pollution-Attitudes | Sadness | Sorrow about the consequences of marine plastic pollution | 21 | 17 | 10 | 7 |
| Marine Plastic Pollution-Attitudes | The need to protect the sea | The motivation associated with communities’ management towards marine protection | 14 | 11 | 5 | 6 |
| Marine Plastic Pollution-Attitudes | Concern for future generations | Feeling of worry about future generations and the possibility of them not using the sea | 12 | 8 | 6 | 2 |
| Stakeholders | Authorities | Government, municipalities, Ministry of Environment, Peruvian navy, Chilean senate, | 73 | 34 | 18 | 16 |
| Stakeholders | Involved in the problem | Stakeholders that have a role in the causes of marine plastic pollution | 94 | 29 | 15 | 14 |
| Stakeholders | Involved in the solution | Stakeholders that have the responsibility to address marine plastic pollution | 88 | 29 | 16 | 13 |
| Stakeholders | Companies | Companies that produce plastic or do economic activities at sea | 75 | 27 | 9 | 18 |
| Stakeholders | Collective Involvement | The participation of citizens, institutions, companies and authorities in significant change towards environmental protection | 69 | 25 | 11 | 14 |
| Stakeholders | Municipalities | Municipalities with jurisdiction in regions with a body of water | 35 | 20 | 12 | 8 |
| Stakeholders | Users | Bathers, sportspersons, and people who visit the sea for leisure | 38 | 20 | 13 | 7 |
| Stakeholders | Fishermen | Group of people whose economic activity is fishing and thus have responsibility of their materials and marine biodiversity | 30 | 15 | 7 | 8 |
| Stakeholders | Constitutional Convention | Constituent body in charge of drafting the new Political Constitution of Chile | 6 | 5 | 0 | 5 |
| Stakeholders | Coastal communities | Coastal communities, coastal indigenous communities | 11 | 4 | 0 | 4 |
| Individual Solution Initiatives | Reduce plastic consumption | Use of biodegradable products and avoidance of plastic consumption in everyday life | 45 | 23 | 10 | 13 |
| Individual Solution Initiatives | Pick up beach litter | Picking up waste from the beach when visiting | 38 | 22 | 15 | 7 |
| Individual Solution Initiatives | Marine Pollution Visibility | Learn and share the information about marine plastic pollution | 44 | 21 | 8 | 13 |
| Individual Solution Initiatives | Intention of Environmental Behavior | The intention to involve in activities that address marine plastic pollution | 14 | 13 | 7 | 6 |
| Individual Solution Initiatives | Recycle | Collection, segregation and classification of waste for recycling | 17 | 11 | 5 | 6 |
| Individual Solution Initiatives | Call out | Disapprove polluting behavior and inform about the consequences of marine plastic pollution | 13 | 10 | 6 | 4 |
| Individual Solution Initiatives-  Beach clean ups | Effective Beach clean ups | Beach clen ups are considered effective for reducing marine plastic pollution | 42 | 22 | 12 | 10 |
| Individual Solution Initiatives-  Beach clean ups | Ineffective Beach clean ups | Beach clen ups are considered ineffective for reducing marine plastic pollution | 14 | 13 | 11 | 2 |
| Structural Solution Initiatives | Environmental Education | Promotion and visibility of environmental protection, active participation in marine protection | 65 | 29 | 14 | 15 |
| Structural Solution Initiatives | Establishment of Laws and Sanctions | The passing of laws that regulate and sanction polluting behavior | 55 | 27 | 15 | 12 |
| Structural Solution Initiatives | Improve Waste Management | Intensify and improve waste management | 24 | 19 | 13 | 6 |
| Structural Solution Initiatives | Revalorization of the sea | The acknowledgement of the importance of the sea and marine life, and the prioritization of marine protection in a societal and governmental level | 30 | 19 | 8 | 11 |
| Structural Solution Initiatives | Institutional support for Pro-Environmental Initiatives | The funding and promotion of pro-environmental initiatives by companies, authorities or NGOs | 12 | 11 | 3 | 8 |
| Structural Solution Initiatives | Political involvement | Political participation towards marine protections, this includes voting for representatives interested in environmental protection and organizing the community in protests | 20 | 11 | 5 | 6 |
| Structural Solution Initiatives | Reducing Plastic Fabrication | The reduction of plastic fabrication and its replacement with biodegradable products | 10 | 9 | 2 | 7 |
| Structural Solution Initiatives | Reducing Environmental footprint | The reduction of the environmental impact that companies have on the planet | 10 | 9 | 3 | 6 |
| Structural Solution Initiatives | Academic Production | Produce research with the goal of learning and informing how marine plastic pollution affects nature and how it could be reduced | 13 | 9 | 1 | 8 |
| Structural Solution Initiatives | Intercultural Education | Education that aims to revalue indigenous communities and their relationship with the environment | 5 | 2 | 0 | 2 |

*Note*: Frequency by quote refers to the number of times a category was coded across the total of the interviews. Frequency by participants refers to the number of interviews in which the code was identified at least one time.
